# {2-[(2-Amino­cyclo­hex­yl)imino­meth­yl]phenolato}dioxidovanadium(V)

**DOI:** 10.1107/S1600536812011592

**Published:** 2012-03-24

**Authors:** Xin-Zhi Sun

**Affiliations:** a College of Chemistry and Pharmaceutical Sciences, Qingdao Agricultural University, Qingdao 266109, People’s Republic of China

## Abstract

In the title dioxidovanadium complex, [V(C_13_H_17_N_2_O)O_2_], the V^V^ atom is in a square-based pyramidal coordination: the basal plane is defined by the phenolate O, imine N and amine N atoms of the tridentate Schiff base ligand, and by one oxide O atom. The apical position is occupied by the other oxide O atom. In the crystal, mol­ecules are connected by N—H⋯O and N—H⋯(O,O) hydrogen bonds, forming a tetra­mer.

## Related literature

For related structures and their proporties, see: Agarwal & Prasad (2006 ▶); Chohan & Sumrra (2010 ▶); Huo *et al.* (2004 ▶); Jing *et al.* (2005 ▶); Lodyga-Chruscinska *et al.* (2008 ▶); Xie *et al.* (2004 ▶); Yuan *et al.* (2009 ▶).
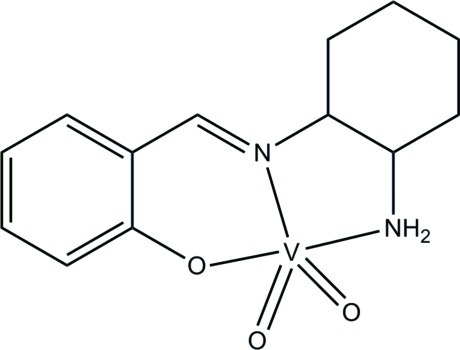


## Experimental

### 

#### Crystal data

[V(C_13_H_17_N_2_O)O_2_]
*M* *_r_* = 300.23
Tetragonal, 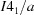

*a* = 19.120 (9) Å
*c* = 15.421 (3) Å
*V* = 5638 (4) Å^3^
*Z* = 16
Mo *K*α radiation
μ = 0.71 mm^−1^
*T* = 298 K
0.23 × 0.20 × 0.20 mm

#### Data collection

Bruker SMART 1000 CCD diffractometer
Absorption correction: multi-scan (*SADABS*; Sheldrick, 2000 ▶) *T*_min_ = 0.854, *T*_max_ = 0.871
19404 measured reflections
2537 independent reflections
1487 reflections with *I* > 2σ(*I*)
*R*_int_ = 0.172

#### Refinement

*R*[*F* ^2^ > 2σ(*F* ^2^)] = 0.099
*wR*(*F* ^2^) = 0.203
*S* = 1.08
2537 reflections
172 parameters
H-atom parameters constrained
Δρ_max_ = 0.44 e Å^−3^
Δρ_min_ = −0.30 e Å^−3^

### 

Data collection: *SMART* (Bruker, 2000 ▶); cell refinement: *SAINT* (Bruker, 2000 ▶); data reduction: *SAINT*; program(s) used to solve structure: *SHELXTL* (Sheldrick, 2008 ▶); program(s) used to refine structure: *SHELXTL*; molecular graphics: *SHELXTL*; software used to prepare material for publication: *SHELXTL*.

## Supplementary Material

Crystal structure: contains datablock(s) I, global. DOI: 10.1107/S1600536812011592/hb6676sup1.cif

Structure factors: contains datablock(s) I. DOI: 10.1107/S1600536812011592/hb6676Isup2.hkl

Additional supplementary materials:  crystallographic information; 3D view; checkCIF report

## Figures and Tables

**Table 1:** Selected bond lengths (Å)

V1—O2	1.607 (5)
V1—O3	1.621 (5)
V1—O1	1.900 (5)
V1—N2	2.095 (5)
V1—N1	2.137 (6)

**Table 2:** Hydrogen-bond geometry (Å, °)

*D*—H⋯*A*	*D*—H	H⋯*A*	*D*⋯*A*	*D*—H⋯*A*
N2—H2*B*⋯O2^i^	0.90	2.26	3.099 (7)	155
N2—H2*B*⋯O3^i^	0.90	2.55	3.229 (7)	133
N2—H2*A*⋯O3^ii^	0.90	2.07	2.948 (7)	166

Symmetry codes: (i) 
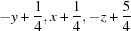
; (ii) 
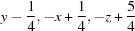
.

**Fig. 1.**
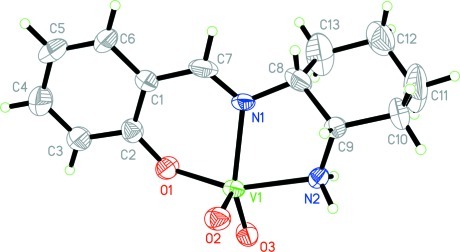
The structure of the title compound, with displacement ellipsoids drawn at the 30% probability level for Non-H atoms.

**Fig. 2.**
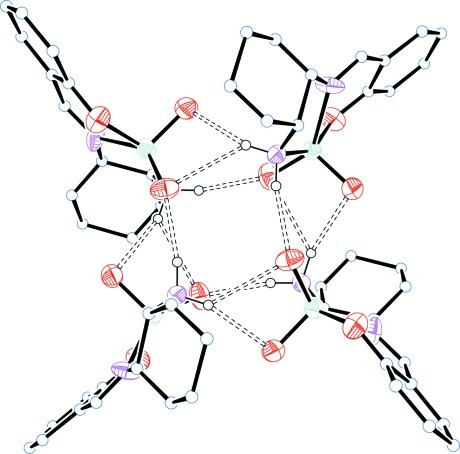
The tetrameric unit in the crystal, with hydrogen bonds shown as double-dashed lines.

## supplementary crystallographic information

### Comment

Oxovanadium complexes with Schiff bases have been received much attention in bioinorganic chemistry (Chohan & Sumrra, 2010; Agarwal & Prasad, 2006; Lodyga-Chruscinska *et al.*, 2008; Yuan *et al.*, 2009). In this paper, the title new dioxovanadium(V) complex, (I), is reported.

In the title dioxovanadium complex, Fig. 1, the V atom is in a square pyramidal coordination. The basal plane of the square pyramid is defined by one phenolate O, one imine N, and one amine N atoms of a Schiff base ligand, and by one oxo O atom. The apical position of the square pyramid is occupied by the other oxo O atom. The bond lengths (Table 1) are comparable to those observed in similar oxovanadium complexes (Xie *et al.*, 2004; Jing *et al.*, 2005; Huo *et al.*, 2004). In the crystal, adjacent molecules are linked through N–H···O and N—H···(O,O) hydrogen bonds (Table 2) to form a tetramer (Fig. 2).

### Experimental

To a MeOH solution (30 ml) of salicylaldehyde (0.122 g, 1.0 mmol) was added a MeOH solution (20 ml) of cyclohexane-1,2-diamine (0.114 g, 1.0 mmol) with stirring. To the above mixture was added a MeOH solution (10 ml) of VO(acac)_2_ (0.265 g, 1.0 mmol) with stirring. The mixture was refluxed for 1 h, affording a clear yellow solution. This was allowed to stand at room temperature for a week and block-shaped single crystals were obtained by slow evaporation.

### Refinement

H atoms attached to C and N atoms were placed in geometrically idealized positions with C*sp*^2^—H = 0.93 Å, C*sp*^3^—H = 0.97–0.98 Å, N—H = 0.90 Å, and constrained to ride on their parent atoms, with *U*_iso_(H) = 1.2*U*_eq_(C and N). Apparent disorder was evident in the cyclohexyl ring.

### Crystal data

**Table:**

[V(C_13_H_17_N_2_O)O_2_]	*D*_x_ = 1.415 Mg m^−^^3^
*M**_r_* = 300.23	Mo *K*α radiation, λ = 0.71073 Å
Tetragonal, *I*4_1_/*a*	Cell parameters from 1125 reflections
*a* = 19.120 (9) Å	θ = 2.7–24.5°
*c* = 15.421 (3) Å	µ = 0.71 mm^−^^1^
*V* = 5638 (4) Å^3^	*T* = 298 K
*Z* = 16	Block, yellow
*F*(000) = 2496	0.23 × 0.20 × 0.20 mm

### Data collection

**Table:**

Bruker SMART 1000 CCD diffractometer	2537 independent reflections
Radiation source: fine-focus sealed tube	1487 reflections with *I* > 2σ(*I*)
Graphite monochromator	*R*_int_ = 0.172
ω scan	θ_max_ = 25.5°, θ_min_ = 1.7°
Absorption correction: multi-scan (*SADABS*; Sheldrick, 2000)	*h* = −23→23
*T*_min_ = 0.854, *T*_max_ = 0.871	*k* = −23→23
19404 measured reflections	*l* = −18→18

### Refinement

**Table:**

Refinement on *F*^2^	Primary atom site location: structure-invariant direct methods
Least-squares matrix: full	Secondary atom site location: difference Fourier map
*R*[*F*^2^ > 2σ(*F*^2^)] = 0.099	Hydrogen site location: inferred from neighbouring sites
*wR*(*F*^2^) = 0.203	H-atom parameters constrained
*S* = 1.08	*w* = 1/[σ^2^(*F*_o_^2^) + (0.0708*P*)^2^ + 16.0234*P*] where *P* = (*F*_o_^2^ + 2*F*_c_^2^)/3
2537 reflections	(Δ/σ)_max_ < 0.001
172 parameters	Δρ_max_ = 0.44 e Å^−^^3^
0 restraints	Δρ_min_ = −0.30 e Å^−^^3^

### Special details

**Table:**

Geometry. All e.s.d.'s (except the e.s.d. in the dihedral angle between two l.s. planes) are estimated using the full covariance matrix. The cell e.s.d.'s are taken into account individually in the estimation of e.s.d.'s in distances, angles and torsion angles; correlations between e.s.d.'s in cell parameters are only used when they are defined by crystal symmetry. An approximate (isotropic) treatment of cell e.s.d.'s is used for estimating e.s.d.'s involving l.s. planes.
Refinement. Refinement of *F*^2^ against ALL reflections. The weighted *R*-factor *wR* and goodness of fit *S* are based on *F*^2^, conventional *R*-factors *R* are based on *F*, with *F* set to zero for negative *F*^2^. The threshold expression of *F*^2^ > σ(*F*^2^) is used only for calculating *R*-factors(gt) *etc*. and is not relevant to the choice of reflections for refinement. *R*-factors based on *F*^2^ are statistically about twice as large as those based on *F*, and *R*- factors based on ALL data will be even larger.

### Fractional atomic coordinates and isotropic or equivalent isotropic displacement parameters (Å^2^)

**Table:**

	*x*	*y*	*z*	*U*_iso_*/*U*_eq_	
V1	0.04762 (6)	0.08630 (6)	0.66104 (7)	0.0417 (4)	
N1	0.1074 (3)	0.0188 (3)	0.5792 (4)	0.0574 (17)	
N2	0.0524 (3)	0.1379 (3)	0.5413 (3)	0.0424 (14)	
H2A	0.0122	0.1616	0.5337	0.051*	
H2B	0.0872	0.1695	0.5436	0.051*	
O1	0.0854 (3)	0.0299 (3)	0.7510 (3)	0.0589 (14)	
O2	−0.0330 (2)	0.0631 (2)	0.6543 (3)	0.0581 (14)	
O3	0.0526 (3)	0.1602 (2)	0.7122 (3)	0.0596 (14)	
C1	0.1142 (4)	−0.0770 (4)	0.6803 (5)	0.062 (2)	
C2	0.0962 (4)	−0.0379 (4)	0.7536 (5)	0.057 (2)	
C3	0.0907 (5)	−0.0727 (5)	0.8315 (6)	0.078 (3)	
H3	0.0788	−0.0477	0.8811	0.093*	
C4	0.1022 (6)	−0.1422 (6)	0.8374 (7)	0.109 (4)	
H4	0.0984	−0.1643	0.8909	0.130*	
C5	0.1195 (6)	−0.1808 (5)	0.7646 (8)	0.111 (4)	
H5	0.1271	−0.2287	0.7692	0.133*	
C6	0.1251 (5)	−0.1488 (5)	0.6875 (7)	0.094 (3)	
H6	0.1365	−0.1748	0.6384	0.112*	
C7	0.1238 (4)	−0.0435 (5)	0.5984 (5)	0.073 (3)	
H7	0.1441	−0.0701	0.5546	0.087*	
C8	0.1201 (5)	0.0398 (6)	0.4903 (6)	0.094 (3)	
H8	0.1107	−0.0015	0.4546	0.112*	
C9	0.0635 (4)	0.0936 (4)	0.4666 (5)	0.063 (2)	
H9	0.0202	0.0670	0.4586	0.075*	
C10	0.0774 (7)	0.1306 (6)	0.3813 (6)	0.110 (4)	
H10A	0.0630	0.0998	0.3346	0.132*	
H10B	0.0478	0.1717	0.3788	0.132*	
C11	0.1431 (8)	0.1505 (8)	0.3664 (10)	0.167 (7)	
H11A	0.1534	0.1903	0.4032	0.200*	
H11B	0.1465	0.1661	0.3067	0.200*	
C12	0.1999 (6)	0.0933 (7)	0.3823 (7)	0.115 (4)	
H12A	0.1972	0.0579	0.3373	0.137*	
H12B	0.2461	0.1141	0.3804	0.137*	
C13	0.1873 (5)	0.0597 (7)	0.4717 (8)	0.134 (5)	
H13A	0.2170	0.0187	0.4764	0.161*	
H13B	0.2023	0.0926	0.5158	0.161*	

### Atomic displacement parameters (Å^2^)

**Table:**

	*U*^11^	*U*^22^	*U*^33^	*U*^12^	*U*^13^	*U*^23^
V1	0.0364 (7)	0.0422 (8)	0.0467 (7)	0.0001 (6)	0.0060 (6)	−0.0070 (6)
N1	0.056 (4)	0.066 (4)	0.050 (4)	0.032 (3)	0.012 (3)	0.003 (3)
N2	0.033 (3)	0.039 (3)	0.055 (4)	−0.001 (3)	0.000 (3)	0.006 (3)
O1	0.072 (4)	0.055 (3)	0.049 (3)	0.009 (3)	−0.008 (3)	−0.004 (3)
O2	0.054 (3)	0.049 (3)	0.071 (4)	−0.002 (2)	0.009 (3)	−0.005 (3)
O3	0.082 (4)	0.047 (3)	0.050 (3)	−0.010 (3)	0.008 (3)	−0.007 (2)
C1	0.076 (6)	0.061 (5)	0.048 (5)	0.010 (4)	0.013 (4)	−0.001 (4)
C2	0.057 (5)	0.064 (6)	0.050 (5)	0.002 (4)	−0.003 (4)	0.005 (4)
C3	0.105 (7)	0.073 (6)	0.055 (6)	0.018 (5)	0.010 (5)	0.002 (5)
C4	0.160 (11)	0.085 (8)	0.080 (7)	0.024 (7)	0.023 (7)	0.025 (7)
C5	0.167 (11)	0.051 (6)	0.115 (10)	0.037 (6)	0.030 (8)	0.003 (6)
C6	0.150 (10)	0.055 (6)	0.076 (7)	0.040 (6)	0.022 (6)	0.015 (5)
C7	0.073 (6)	0.097 (7)	0.048 (5)	0.044 (5)	0.005 (4)	−0.012 (5)
C8	0.095 (8)	0.124 (8)	0.062 (6)	0.045 (6)	0.034 (5)	0.014 (6)
C9	0.083 (6)	0.063 (5)	0.043 (4)	0.028 (5)	0.003 (4)	0.002 (4)
C10	0.161 (12)	0.115 (9)	0.054 (6)	0.037 (8)	0.029 (7)	0.036 (6)
C11	0.142 (13)	0.200 (16)	0.158 (13)	−0.015 (12)	0.024 (10)	0.123 (12)
C12	0.111 (9)	0.135 (10)	0.098 (8)	−0.012 (8)	0.034 (7)	0.026 (7)
C13	0.058 (7)	0.215 (14)	0.129 (10)	0.002 (7)	−0.005 (7)	0.079 (10)

### Geometric parameters (Å, º)

**Table:**

V1—O2	1.607 (5)	C5—H5	0.9300
V1—O3	1.621 (5)	C6—H6	0.9300
V1—O1	1.900 (5)	C7—H7	0.9300
V1—N2	2.095 (5)	C8—C13	1.370 (12)
V1—N1	2.137 (6)	C8—C9	1.536 (11)
N1—C7	1.267 (9)	C8—H8	0.9800
N1—C8	1.448 (10)	C9—C10	1.517 (11)
N2—C9	1.447 (8)	C9—H9	0.9800
N2—H2A	0.9000	C10—C11	1.332 (15)
N2—H2B	0.9000	C10—H10A	0.9700
O1—C2	1.314 (8)	C10—H10B	0.9700
C1—C6	1.394 (11)	C11—C12	1.561 (17)
C1—C2	1.398 (10)	C11—H11A	0.9700
C1—C7	1.428 (11)	C11—H11B	0.9700
C2—C3	1.377 (11)	C12—C13	1.541 (13)
C3—C4	1.350 (12)	C12—H12A	0.9700
C3—H3	0.9300	C12—H12B	0.9700
C4—C5	1.383 (13)	C13—H13A	0.9700
C4—H4	0.9300	C13—H13B	0.9700
C5—C6	1.342 (13)		
			
O2—V1—O3	109.2 (3)	N1—C7—H7	116.7
O2—V1—O1	104.8 (2)	C1—C7—H7	116.7
O3—V1—O1	96.7 (2)	C13—C8—N1	115.6 (9)
O2—V1—N2	96.6 (2)	C13—C8—C9	115.2 (9)
O3—V1—N2	90.9 (2)	N1—C8—C9	107.0 (6)
O1—V1—N2	153.4 (2)	C13—C8—H8	106.1
O2—V1—N1	108.0 (2)	N1—C8—H8	106.1
O3—V1—N1	141.5 (3)	C9—C8—H8	106.1
O1—V1—N1	83.4 (2)	N2—C9—C10	116.3 (7)
N2—V1—N1	75.0 (2)	N2—C9—C8	107.8 (6)
C7—N1—C8	116.1 (7)	C10—C9—C8	113.3 (7)
C7—N1—V1	124.2 (5)	N2—C9—H9	106.2
C8—N1—V1	118.8 (5)	C10—C9—H9	106.2
C9—N2—V1	115.6 (4)	C8—C9—H9	106.2
C9—N2—H2A	108.4	C11—C10—C9	116.6 (11)
V1—N2—H2A	108.4	C11—C10—H10A	108.1
C9—N2—H2B	108.4	C9—C10—H10A	108.1
V1—N2—H2B	108.4	C11—C10—H10B	108.1
H2A—N2—H2B	107.4	C9—C10—H10B	108.1
C2—O1—V1	129.9 (5)	H10A—C10—H10B	107.3
C6—C1—C2	119.9 (8)	C10—C11—C12	115.4 (11)
C6—C1—C7	119.5 (8)	C10—C11—H11A	108.4
C2—C1—C7	120.5 (7)	C12—C11—H11A	108.4
O1—C2—C3	119.4 (7)	C10—C11—H11B	108.4
O1—C2—C1	122.8 (7)	C12—C11—H11B	108.4
C3—C2—C1	117.9 (8)	H11A—C11—H11B	107.5
C4—C3—C2	121.4 (9)	C13—C12—C11	108.9 (10)
C4—C3—H3	119.3	C13—C12—H12A	109.9
C2—C3—H3	119.3	C11—C12—H12A	109.9
C3—C4—C5	120.7 (10)	C13—C12—H12B	109.9
C3—C4—H4	119.7	C11—C12—H12B	109.9
C5—C4—H4	119.7	H12A—C12—H12B	108.3
C6—C5—C4	119.7 (9)	C8—C13—C12	116.7 (9)
C6—C5—H5	120.1	C8—C13—H13A	108.1
C4—C5—H5	120.1	C12—C13—H13A	108.1
C5—C6—C1	120.4 (9)	C8—C13—H13B	108.1
C5—C6—H6	119.8	C12—C13—H13B	108.1
C1—C6—H6	119.8	H13A—C13—H13B	107.3
N1—C7—C1	126.6 (7)		

### Hydrogen-bond geometry (Å, º)

**Table:**

*D*—H···*A*	*D*—H	H···*A*	*D*···*A*	*D*—H···*A*
N2—H2*B*···O2^i^	0.90	2.26	3.099 (7)	155
N2—H2*B*···O3^i^	0.90	2.55	3.229 (7)	133
N2—H2*A*···O3^ii^	0.90	2.07	2.948 (7)	166

Symmetry codes: (i) −*y*+1/4, *x*+1/4, −*z*+5/4; (ii) *y*−1/4, −*x*+1/4, −*z*+5/4.
